# From the Editor’s Desk

**Published:** 2014

**Authors:** PJ Commerford

**Affiliations:** Editor-in-Chief

Food and the eating thereof is a universal part of the human condition, and life is obviously impossible without adequate nutrition. The fact that the composition of diets varies so dramatically across the globe and that human populations survive and thrive fairly successfully despite seemingly very different nutrient intakes provides food for thought! Either the exact composition of the diet is irrelevant or else humans are biologically remarkably adaptable to great variation in the composition of their diet. If either of the statements in the previous sentence is correct then those individuals in what I refer to as the ‘diet industry’, an industry with enormous media appeal and reward, should feel seriously threatened.

In light of the dramatic advances in the pharmacological and interventional management of patients with cardiovascular diseases, it is unfortunate that apparently conflicting advice on such a simple matter as diet is offered to patients and those at risk of disease. Such conflicting advice surely confuses rather than educates those at whom it is aimed. Lifestyle and diet are the topics reviewed by Opie (page 298) in an in-depth and scholarly review, which addresses in a balanced manner many of the current controversies. The accompanying editorial from Raal (page 302) is further valuable commentary.

Awad and colleagues (page 269) report on the high prevalence rates of hypertension in the Gambia and Sierra Leone, as has been previously reported from other parts of Africa. The South African hypertension practice guideline prepared by the Hypertension Guideline Working Group of the Southern African Hypertension Society is published on page 288. It is comprehensive and includes information on lifestyle modification and education, in addition to detailed advice on pharmacotherapy. An accompanying comment (page 296) addresses the value and importance of such guidelines in clinical practice. It would be interesting to hear comment from colleagues in other parts of Africa as to the applicability and relevance of these guidelines to practice in their own countries.

Otaigbe and colleagues (page 265) report on the prevalence of congenital heart disease, detected by echocardiography, among children referred to two specialist paediatric cardiology clinics in the Niger Delta region of Nigeria. Such information adds to our increasing knowledge of patterns of cardiovascular disease in Africa. It must be borne in mind however that this is not a population-based study, referral bias remains possible, and the authors’ attribution of the high prevalence to environmental pollution is speculative.

Despite the success of percutaneous interventions, coronary artery bypass grafting is still a very common operation and the impact of interventions and risk factors for complications continues to be investigated. Cingoz and colleagues (page 279) examine the impact of co-morbidity on bleeding after the operation, while Yildiz and co-workers (page 259) examine the value of patient-directed education on patient anxiety after surgery. The importance of the anxiety experienced by patients and families of hospital survivors of major cardiac surgery is often underestimated by healthcare professionals.

